# Modelling animal movement as Brownian bridges with covariates

**DOI:** 10.1186/s40462-019-0167-3

**Published:** 2019-06-25

**Authors:** Bart Kranstauber

**Affiliations:** 1 Department of Evolutionary Biology and Environmental Studies, University of Zurich, Winterthurerstrasse 190, Zurich, CH-8057 Switzerland; 2 Kalahari Meerkat Project, Kuruman River Reserve, P.O. Box 64, Van Zylsrus, 8467 Northern Cape South Africa

**Keywords:** Animal tracking, Brownian bridge covariates model, Brownian bridge movement model, Meerkats, Movement ecology, *Suricata suricatta*, Utilization distributions

## Abstract

**Background:**

The ability to observe animal movement and possible correlates has increased strongly over the past decades. Methods to analyze trajectories have developed in parallel, but many tools fail to make an immediate connection between a movement model, covariates of the movement, and animal space use.

**Methods:**

Here I develop a novel method based on the Brownian Bridge Movement Model that facilitates investigating and testing covariates of movement. The model makes it possible to flexibly investigate different covariates including, for example, periodic movement patterns.

**Results:**

I applied the Brownian Bridge Covariates Model (BBCM) to simulated trajectories demonstrating its ability to reproduce the parameters used for the simulation. I also applied the model to a GPS trajectory of a meerkat, showing its application to empirical data. The value of the model was shown by testing the interaction between maximal daily temperature and the daily movement pattern.

**Conclusion:**

This model produces accurate parameter estimates for covariates of the movements and location error in simulated trajectories. Application to the meerkat trajectory also produced plausible parameter estimates. This new method opens the possibility to directly test hypotheses about the influence of covariates on animal movement while linking these to space-use estimates.

## Background

Through movement, animals connect the world: animal movements are key to understanding important behavioural, ecological and evolutionary processes such as migration, social interactions, nutrient fluxes, disease spread, seed dispersal and gene flow [1]. Although there is a long history of studying movement and space use, going back to the early work of Burt’s [2] study on home ranges and territoriality, recent technological developments have made it possible to conduct studies over a wide range of spatial and temporal scales and with resolutions previously unimaginable across a variety of species (e.g. [3–5]).

The analysis of animal tracking data is currently undergoing an exciting transformation. Classical tools for estimation of space use and utilization distributions (UDs) such as minimum convex polygons or kernel density surfaces rely on an independent sample of positions of the individual [6]. These assumptions are clearly not valid especially for modern data collection technologies, where trajectories are typically sampled with high frequencies, often producing several locations per hour. As a result, these methods produce inaccurate estimates of territory sizes and borders. With such highly resolved trajectories it further becomes important to account for the accuracy of the tracking device, although most classical methods are not able to do so.

Newly developed tools are more suitable for highly resolved trajectories; they take advantage of the positional correlations instead of removing and/or ignoring them [7]. These new tools make use of various underlying movement models, such as Brownian bridge (e.g. [8–10]) and biased random bridge [11]. The dynamic Brownian Bridge Movement Model is, for example, useful for calculating the area used by animals, while accounting for behavioural changes in the movement patterns. This method though requires the behavioural changes to be infrequent relative to the sampling frequency of the trajectory. By analyzing the track as a continuous-time stochastic process [7, 12], an accurate description of the movement process and home range can be obtained.

However, most current approaches lack a natural connection to analyze the trajectory with respect to covariates. One notable exception is the work of Wilson et al. [13] who integrate environmental covariates using a spatially discrete model. Other methods have focused more on analyzing the trajectory as such, without focusing on space use, by directly calculating metrics from the track and comparing these to covariates (e.g. wind and migration speed, [14, 15]). More recently step-selection functions have become popular; here trajectories are sampled to regular intervals and each step is compared to a set of alternative steps that are generated using logistic regressions [16, 17]. This method has the advantage that it easily incorporates many different covariates of movement, but it requires regularized trajectories and there is no natural relationship to space use [18]. Additionally, step-selection functions do not account for measurement errors, where the actual location of the animal is not the same as the reported position. In cases where animals move large distances combined with the accuracy of modern tracking technology (e.g. GPS) location errors can sometimes be reasonably ignored. However, for slower moving animals with frequent location recordings, measurement error needs to be accounted for to derive useful information from tracking data [19]. Similarly, the inclusion of location errors is important for other tracking methodologies such as geolocation and the Argos system that have considerable location errors [20–22].

Nearly all animals show some regular or environmentally induced variation in movement patterns. Animals commonly have a periodic movement activity: usually studied are daily (e.g., nocturnal, diurnal, or crepuscular activity) and yearly (e.g., altitudinal or long-distance migrations) patterns (e.g. [23–25]). Less frequently studied are movement patterns that relate to the lunar cycle (e.g. [26, 27]). There have been various ways to account for these periodic variations in movement. Approaches based on the Brownian Bridge Movement Model account for behaviourally heterogeneous trajectories but do not relate temporal variation to a specific period [9, 10]. These methods rely on a moving window approach, therefore it is difficult to describe patterns when observations are infrequent relative to the period. Alternatively, continuous time movement models can include periodic movement patterns [28]. A more general approach is to fit a model with a smoothing term to investigate movement over the day (e.g. [29]). The advantage of using smoothing terms is that it gives a formal function that can be used to fit interactions with other factors influencing the movement pattern over the day.

Here I develop a method that calculates space use taking into account movement properties and covariates. This model generalizes the Brownian Bridge Movement Model and considers factors influencing both the movement and location error. Animal movement models based on Brownian bridges have found their merits despite the limiting assumptions as isotropic movement and not being bound to ranges. The nature of the covariates influencing movement and location error can be flexible, including the influence of important ecological and social factors. This model, termed the Brownian Bridge Covariates Model (BBCM), is able to account for errors in the observation process and fit covariates of these errors, ultimately leading to a more accurate and predictive model. First, I evaluate the model by estimating known parameters from simulated trajectories. Second, I apply this model to a trajectory of a meerkat (*Suricata suricatta*), to investigate changes to the circadian movement pattern under the influence of weather conditions.

## Methods

This section is divided into three parts: description of the method, validation of the model by applying it to simulated tracks, and use of this method on real tracking data of a meerkat. All these analyses were conducted in R [30].

### Brownian bridges including covariates

Pozdnyakov et al. [31] identified that the distances between observations have the following covariance matrix:

$$\mathbf{\Sigma}_{X} = \left[\begin{array}{ccccc} \sigma^{2}\uptau_{1} + 2\delta^{2} & -\delta^{2} & 0 & \cdots & 0 \\ -\delta^{2} & \sigma^{2}\uptau_{2} + 2\delta^{2} & -\delta^{2} & \cdots & 0 \\ 0 & -\delta^{2} & \sigma^{2}\uptau_{3} + 2\delta^{2} &\cdots & 0 \\ \vdots & \vdots & \vdots & \ddots & \vdots \\ 0 & 0 & 0 & \cdots & \sigma^{2}\uptau_{n} + 2\delta^{2} \end{array}\right] $$

Where *n* is the number of intervals in a trajectory consisting of *n*+1 locations, *τ*_*i*_=*t*_*i*+1_−*t*_*i*_ is the time interval between locations, *σ*^2^ the Brownian motion movement variance and *δ*^2^ the measurement error variance. Building on these methods, it is possible to fit covariates to both the movement trajectory and measurement errors. First, the covariance matrix needs to be adjusted as follows, the measurement error for each location and motion variance for each segment need to be separated. In that case **Σ**_*X*_ can be formed based on $\boldsymbol {\sigma }^{2}=\left (\sigma _{1}^{2},\ldots,\sigma _{n}^{2}\right)$ and $\boldsymbol {\delta }^{2}=\left (\delta _{1}^{2},\ldots,\delta _{n+1}^{2}\right)$. This creates the following covariance matrix.

$$\mathbf{\Sigma}_{X} \,=\,\! \left[\begin{array}{ccccc} \!\sigma_{1}^{2}\uptau_{1} \,+\, {\delta_{1}^{2}\,+\,\delta_{2}^{2} }& -\delta_{2}^{2} & 0 &\cdots & 0 \\ -\delta_{2}^{2} & \!\sigma_{2}^{2}\uptau_{2} \,+\,{ \delta_{2}^{2}\,+\, \delta_{3}^{2} }& -\delta_{3}^{2} & \cdots & 0 \\ 0 & -\delta_{3}^{2} & \!\sigma_{3}^{2}\uptau_{3} \,+\,{ \delta_{3}^{2}\,+\, \delta_{4}^{2} }&\cdots & 0 \\ \vdots & \vdots & \vdots & \ddots & \vdots \\ 0 & 0 & 0 & \cdots & \sigma_{n}^{2}\uptau_{n} \,+\, {\delta_{n}^{2} \,+\, \delta_{n+1}^{2}} \end{array}\right] $$

To generate ***σ***^2^ and ***δ***^2^ I can rely on frequently used modelling tools where a design matrix is multiplied by a vector of coefficients. For this reason it is important to model the movement and location-error variances since they are cumulative in contrast to standard deviations. This means ***σ***^2^ with *m* covariates can be generated as follows.

$$\left[\begin{array}{c} \sigma_{1}^{2} \\ \sigma_{2}^{2} \\ \sigma_{3}^{2} \\ \vdots \\ \sigma_{n}^{2} \end{array}\right]=\left[\begin{array}{cccc}x_{1,1}&x_{1,2}&\dots&x_{1,m}\\ x_{2,1}&x_{2,2}&\dots&x_{2,m}\\ x_{3,1}&x_{3,2}&\dots&x_{3,m}\\ \vdots & \vdots &\ddots& \vdots \\ x_{n,1}&x_{n,2}&\dots&x_{n,m} \end{array}\right] \left[\begin{array}{c} \upbeta_{\sigma^{2},1}\\ \upbeta_{\sigma^{2},2}\\ \vdots \\ \upbeta_{\sigma^{2},m}\end{array}\right] $$

Similarly, ***δ***^2^ can be generated based on the location-error covariates. Using this strategy and the likelihood equation given by Pozdnyakov et al. [31], now the likelihood can be evaluated based on $\phantom {\dot {i}\!}\boldsymbol \upbeta _{\sigma ^{2}}$ and $\phantom {\dot {i}\!}\boldsymbol \upbeta _{\delta ^{2}}$. Here I chose to evaluate this equation using Markov chain Monte Carlo (MCMC) methods. To do this I relied on the stan library accessed through the rstan R package [32]; for computational efficiency all covariates were scaled. Through this library, direct optimization of the parameters is also possible. For all model fits I evaluated the trace plots to confirm that all chains are well mixed and the warmup period is sufficiently long. I report maximal $\hat {R}$ values to asses model fit [33]; values above 1.1 indicate a poor model fit.

#### Estimate origin of the trajectory

The original estimation of *σ* and *δ* described by Pozdnyakov et al. [31] makes the assumption that the origin of the trajectory is located at 0,0 and thus known. Since parameter estimation depends on the distances between locations, this has no consequences for the parameter estimation. In most tracking studies this assumption, that the starting location is known, is difficult to justify. Often the release location of the animal is recorded with similar accuracy as the other locations in the track. Furthermore, the first recording by an animal-borne tracking device is generally not recorded at the same time as the release. Therefore I expanded earlier analysis by including the estimation of the first location; knowing the start of the trajectory is important for calculating the location of the UD in space. Estimation of the first location can be achieved by extending the likelihood function to include the likelihood of this location. The distance between the first location and the first observed location has a variance of $\delta _{1}^{2}$, while it has covariance with the first step length of $-\delta _{1}^{2}$. This procedure creates the following covariance matrix that still profits from the tribanded nature for efficient model evaluation.

$$\mathbf{\Sigma}_{X} = \left[\begin{array}{cccc} \delta_{1}^{2} &-\delta_{1}^{2}&0&\cdots \\ -\delta_{1}^{2}& \sigma_{1}^{2}\uptau_{1} + {\delta_{1}^{2}+\delta_{2}^{2} }& -\delta_{2}^{2} &\cdots \\ 0& -\delta_{2}^{2} & \sigma_{2}^{2}\uptau_{2} +{ \delta_{2}^{2}+ \delta_{3}^{2} }& \cdots \\ \vdots& \vdots & \vdots & \ddots \\ \end{array}\right] $$

To estimate the likelihood of the combined initial location and increments of the trajectory, the vector containing the expected values also needs to be updated. Previously this vector consisted purely of expected distances between locations, which are zero. To include the first location the deviations from expected can be represented as follows $\mathbf {X}=(Z_{0}-\gamma, X_{1},\ldots,X_{n})^{\intercal }$, where *γ* is the origin of the trajectory.

### Generating covariates

For calculating the design matrix, I utilized readily available R tools, making it possible to profit from the familiar formula specification. For the movement variance, it is important to realize that these coefficients are representative for the complete interval between two locations; covariates thus need to represent the conditions during this period and not conditions at the start or end location.

Special care needs to be taken since variances are modelled; this means all estimates need to be positive. To enforce this, either models with an intercept can be used or models where all values for the first covariate are either completely positive or negative. In both cases the minimal or maximal value for the first parameter depends on the other parameter estimates and can be calculated during sampling. These constraints on the covariates ensure the sampled parameters result in positive variance estimates.

#### Periodic movement covariates

An important covariate of movement I included in the model is the periodic movement pattern. This was done by estimating circular smoothing functions as a covariate of the movement variance. Smooths are flexible tools for describing these patterns without making a priori assumptions. By using circular smoothers there is no need to artificially cut the day or other time periods over which the movement is estimated at a specific time point. I used cubic B splines, which consist of four segments per smoothing term. These splines give us the instantaneous movement rate at any time. Since the $\sigma _{m}^{2}$ values are representative of the movement rate over the whole segment, I needed to derive the average movement rate for each segment. This can be accomplished by integrating the splines over the time period between observations and then dividing it by the time interval. For regular splines this can be achieved using analytical integration, allowing for quick calculations. The disadvantage of this approach is that the intercept is included in the smoothing term. When such a model is combined with an intercept it will have multiple solutions. I used an alternative approach, where I separated out the constants in a model. No attempt has been made to solve this analytically; I relied on the numerical integration of existing implementations [34]. These also give us additional flexibility with the specification of smoothing terms, as it opens the possibility of custom knot placement. Non-regular knot placement is mainly of use when the tracked individual has not been observed for a longer time period when the tags are turned off or not observed during the resting period. Within this time period there are no observations and thus there is no information on the movement pattern. When a uniform distribution of the smoothing terms would have been used, information to estimate the smoothing terms during the period when the GPS was turned off is lacking.

### Validation

To validate the model I first simulated irregular trajectories with various parameters to investigate if the model can accurately estimate parameters without periodic terms. To investigate the accuracy of these estimations I varied the length of the trajectories (*n* = 100, 200, 500, 1000, 2000, 5000 and 10000). For each trajectory length three replicates were calculated. The tracks were simulated including a continuous covariate of both the movement variance and the location error and a discrete covariate with three different levels of both. For each segment, one of three movement states was sampled (*Pr*=[0.5,0.2,0.3]), each state had its own movement rate (*σ*^2^=[0.3,0.8,2]), the continuous movement covariate was sampled uniformly random between 0.0 and 0.5 and had a slope of 2. For the location error, three categories were sampled (*Pr*=[0.25,0.5,0.25]) per location, each category was associated with a specific location error (*δ*^2^=[3,3.9,6]), the continuous error covariate was sampled between -1.0 and 1.0 and had a slope of 0.4. Time intervals were generated by adding one to a gamma distribution (*α*=3,*β*=0.1, mean time lag = 31.06 s). For these trajectories, 10 parameters were estimated in total: four relating to the location error, four relating to the movement, and two for the start location. The model was fitted based on intercepts and treatment contrast with four MCMC chains, each with 5000 iterations of which 2500 were warmup iterations; chains were thinned by retaining every tenth iteration. To assess the importance of incorporating location error I have also fitted these models using only a single intercept to describe the same trajectories.

To investigate the ability of the model to describe periodic patterns I simulated trajectories of a 10-day duration. These trajectories were simulated with five different circadian movement patterns, consisting of either six or ten knots. Models were estimated with the same number of knots as specified for the simulated trajectory. Trajectories included a location error of *δ*^2^=10. To investigate the accuracy of these estimations as a function of sample size I repeated the analysis four times with track lengths varying between 500 and 5000 locations. For optimization I used 5000 MCMC iterations replicated across four chains. For these validations I assess the ability to reproduce known simulation parameters.

### Application to tracking data

I tested the method described above by applying it to a trajectory of a meerkat, which has been followed over 29 days. Meerkats live in social groups ranging from 2 to 50 individuals, where the dominant pair controls most breeding. At the Kalahari Meerkat Project detailed behavioural observations have been conducted for over 20 years (for an overview see [35]). Meerkat groups emerge from their burrows around sunrise and collectively forage predominantly for invertebrate prey while moving. During summer the groups stop moving during the hottest part of the day when they take a break and often rest in the shade. In the afternoon meerkats resume foraging before groups return to a sleeping burrow [36]. The meerkat was fitted with a collar on 25th of January 2018; the collar was removed after three months. At the time of capture the individual weighed 606g. The GPS logger (weight 20 g, Gipsy 5, Technosmart, Rome, Italy) was programmed to take 5 locations with a 1 Hz frequency every 5 minutes for 12 hours a day from 7 am until 7 pm. Outliers were filtered by a set of criteria based on distances and turn angles either between locations or bursts, high speeds between one or more bursts and a high spread of locations within a burst. The criteria used were validated using independent observations of the meerkat group. From the original 16169 locations, 13513 observations were retained. With this dataset, I explored different periodic smoothing to describe the activity over the day. Since there are no observations during the night time I only specified knots during the daytime. The first knot is specified at the first observation across days while the last knot is specified at the last location. The number of knots varied between 5 and 12 to investigate the number of knots producing the best-fitting model. For location error I did not vary the terms; I fitted a model without intercept, taking the squared Horizontal Dilution Of Precision (HDOP) as the only covariate.

The capabilities of the model to test a hypothesis were investigated by estimating the response of the meerkat to maximal daily temperature. Temperature data were derived from the reserve’s weather station and varied between 24.1 ^∘^C and 40.8 ^∘^C during the tracking period. I tested whether meerkats had a reduced foraging and thus movement activity on hot days during the late part of the morning when temperatures rise in comparison to the activity pattern on the other cooler days. As a threshold for hot days I used 35 ^∘^C; this same threshold is used for bird studies in the region [37]. I started using a model with the same number of knots as the model found to best describe the movement. On hot days I duplicated the smoothing terms that peak between 9 and 12 o’clock. In this way the model can describe different movement patterns within the morning time period for hot and cool days.

## Results

### Validation

**Fig. 1:**
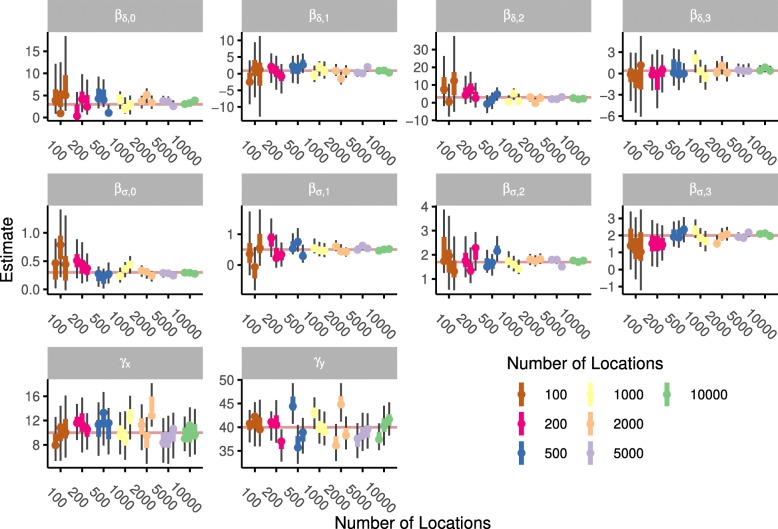
Results of fitting the BBCM to simulated trajectories. Each simulation was based on 10 parameters that were estimated using the model. Each panel displays the estimations for one parameter; the set parameter value is shown by the horizontal line. Simulations were conducted for three replicates of seven different trajectory lengths. Vertical lines indicate 50% and 95% confidence intervals from the MCMC simulation; points reflect the maximum likelihood estimate

The model successfully reproduced the parameters used to simulate trajectories (Fig. 1): the $\hat {R}$ values for all parameters were below 1.009. Confidence intervals of the estimated parameters overlapped with the values used for simulations independent of the length of the simulated trajectory. For these and all other models, parameter estimates are shown in the supplementary material. With increasing trajectory length the confidence interval for the estimated parameter shrinks considerably. The estimated parameters perform equally well for location error as for movement parameters. The benefits of incorporating additional variables to appropriately describe the location error depend on the trajectory length. For trajectories longer than 5000 observations the AIC difference for models incorporating covariates of the location error was always positive (range from 4.4 to 31.9), vice versa for trajectories shorter than 200 all AIC differences were negative (range from -7.8 to -1.4). To assess the influence of error modelling on the fit of movement parameters I calculated the variance of the difference between the simulated values and the MCMC chains. The variance was higher for 19 out of 28 parameters in models without error modelling.

**Fig. 2:**
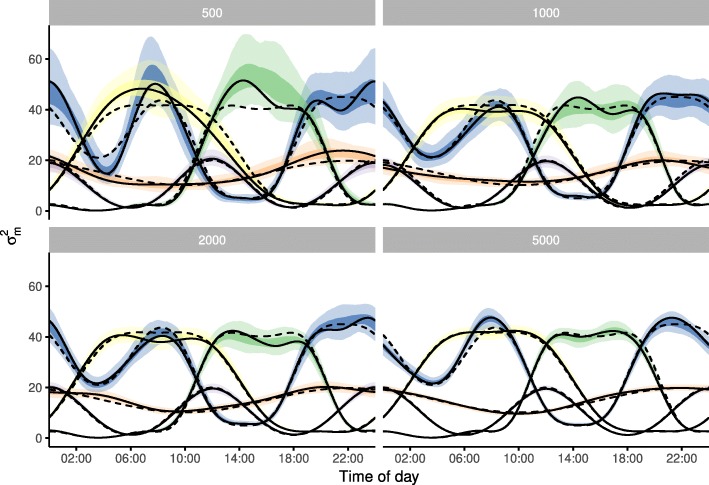
Results from fitting daily movement smoothers to test trajectories. I simulated four daily movement patterns; for each pattern four different trajectory lengths have been simulated, this length is indicated in the subpanel header. Each daily movement pattern is shown in a different colour. The model fit to the simulated trajectories is visualized by the maximum likelihood estimate as a solid line with the 50% and 95% confidence intervals shown by the darker and lighter shading, respectively. Dashed lines depict the simulated daily movement patterns

I found that simulated daily movement patterns were well captured by the model (Fig. 2, $\hat {R}< 1.013$). This fit is independent of the number of changes in the pattern. Even at the smallest track length of 500 the general features of the movement patterns are retained. With increasing sample size the confidence intervals become more narrow and still include the simulated pattern.

### Application to tracking data

**Fig. 3:**
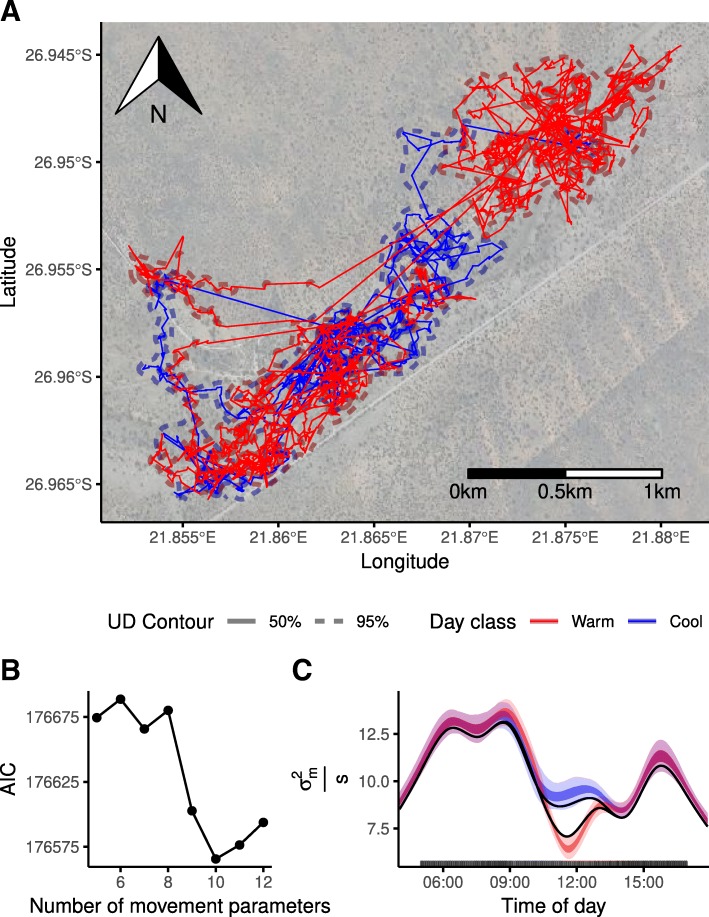
Results of fitting the BBCM to a meerkat trajectory. **a** | The trajectory of the meerkat studied; track colour reflects whether the day was classified as cool or hot. The isolines reflect the 50% and 95% contour of the UD calculated separately for cooler and warmer days. **b** | The AIC of the various models fitted to the trajectory as a function of the number of knots. AIC drops until ten knots and then increases slightly. **c** | A plot of the fitted activity pattern that includes the effect of the cooler days. The graph shows the maximum likelihood estimate (black line) and 50% and 95% confidence intervals by the darker and lighter shade, respectively

I successfully fitted models to the meerkat trajectory (Fig. 3a). The model fit strongly depended on the number of knots; models with the lowest AIC used ten knots (Fig. 3b, for all models $\hat {R}<1.007$). As the smoothers form a circular approximation of the activity, the movement pattern throughout the day can be calculated. During the night the confidence interval was much wider since there are no observations at night; the variation in movement during the night cannot be described. The average estimated $\sigma _{m}^{2}$ during the night is 0.85 *m*^2^/*s* which is much lower than the daily average of 8.19 *m*^2^/*s*.

On 22 out of 29 days the air temperature rose above 35 ^∘^C. When comparing the model taking hot days into account, the AIC drops by 35.7 points compared to the reference model; parameters of this model have a $\hat {R}$ value below 1.007. Visualization of these model outputs showed that during the cooler days there was more movement in the late morning compared to the warmer days (Fig. 3c).

## Discussion

Results of this study suggest that the described BBCM approach was successful in capturing movement patterns that included covariates. The model worked accurately to describe the relationships between movement and different covariates, independent of whether these covariates were categorical, continuous or a daily movement pattern. A combination of linear and factorial covariates, as well as periodic covariates, have been tested here using simulations. A comparison with models only incorporating one estimate for location error shows that extra parameters accounting for covariates of the location error results in an improved model fit for longer trajectories. I also used this method successfully to investigate the trajectory of a meerkat, to test specific hypotheses about its movement behaviour. Using the proposed BBCM, the observed movement pattern is used to estimate the influence of covariates and periodic movement patterns; these are subsequently integrated into the calculation of the UDs. Models of the movement process and UDs are important for many different subsequent analytical approaches in the field of movement ecology.

The BBCM is computationally efficient because it profits from the tribanded matrix inversion; it can comfortably be used to investigate trajectories with (10,000) locations. Computational time also depends on the number of covariates and mostly on the method of evaluation, either through MCMC or optimization. An interesting alternative is to use Kalman filters, which has been explored by Fleming et al. [38], making it possible to evaluate models efficiently. This approach would also profit from the ability to integrate the persistence of motion. Future work is needed to evaluate this and compare its advantages and disadvantages.

My model is based on covariates of both the location error and the movement process. In the next two sections I will discuss considerations for specific covariates of both.

### Location-error covariates

The location-error covariates are specific to the time the location has been observed. A familiar covariate could be the location accuracy as reported by the GPS, frequently reported as the HDOP. This measurement reports the influence of the geometry of the satellites on the horizontal locational accuracy of the GPS. Even though this measure relates to accuracy it is not directly translatable to error measurement [39]. Therefore modelling error as a covariate is an efficient way to estimate the conversion coefficient. The HDOP does not necessarily relate linearly to the location-error variance. An earlier study has for example modelled it as a linear relationship with the standard deviation of the location error [40] but did not evaluate other possible relations. If the covariate has a linear relationship with its standard deviation it is advisable to transform it by squaring in order to make the relationship with the location-error variance linear. Different transformations of continuous covariates can be explored to identify the one that produces a linear relation with the modelled variances.

Frequently the influence of habitat on location error is discussed. Forests and other covered habitats generally produce larger location errors [19, 41, 42]. Including these is possible if the observed locations are annotated with habitat information. The difficulty is that the habitat at an observed location is not necessarily the same as the habitat on the true position of the animal with the GPS tag. This means that the habitat where the GPS obtained the location with the associated error is not necessarily the one that is modelled. This is likely not very problematic in cases where the habitat is continuous and locations rarely fall close (i.e. within the location error) to an edge of a habitat and thus has a low likelihood to be associated with an incorrect habitat classification. In cases where the habitat is a fine mosaic of different habitat types it might not be possible to include habitat reliably without further research.

Frequently animals are tracked using a combination of technologies. A well-known example is GPS-Argos tracking that produces both location estimates through Argos and GPS. On other occasions animals are tracked using a combination of visual observations and by technological means. Including tracking technology as a covariate in a model for location error is a viable approach to account for differing location errors produced by different tracking technologies.

### Movement variance covariates

Covariates of movement require slightly more consideration compared to the location-error covariates. Since these are representative for the time periods between the observations they need to be summarized for this time. Besides periodic movement, one likely covariate for the movement rate is the acceleration as measured by modern tags. If acceleration is to be included as a covariate it needs to be averaged over the entire segment between two observations. This could be achieved by including time-weighted averaged Overall Dynamic Body Acceleration (ODBA) as an index of activity. It has been found that ODBA is an accurate predictor of movement speed [43], and thus it could be an important covariate. A second solution could be to classify acceleration bursts as either active or non-active and use the proportion of active acceleration burst as a covariate (e.g. [11]). Taking into account this covariate becomes especially important if sampling is dependent on acceleration as is done with acceleration-informed GPS sampling (i.e. [44]).

The BBCM is centred around temporally varying covariates; these are by no means the only important factor to understand space use and movement of animals. A large class of covariates that have not been integrated here are spatially distributed environmental variables (e.g. habitat type and elevation). This class of covariates also include barriers to movement that can not be crossed; these range from fences to shorelines. An important contribution has recently been made by Wilson et al. [13] who develop a model to analyse movement while incorporating environmental covariates. Their model depends on a discretization of space in contrast to the BBCM that treats space as continuous. The later has the advantage that probabilities for any position in space can be calculated directly. Work in the near future should focus on integrating both approaches where different kinds of covariates can be easily combined, preferably in continuous space.

## Conclusion

Using the BBCM it is possible to directly test hypotheses concerning the movement patterns of animals, taking into account observation errors and other covariates. My approach is based on the existing rstan library for model evaluation, making it possible to profit from existing tools in the R environment. By using these tools new models can be implemented and evaluated efficiently to allow for flexible hypothesis testing.

## Data Availability

The model developed here is available in the R package “moveBrownianModel” (https://gitlab.com/bartk/moveBrownianModel).

## Abbreviations

BBCM: Brownian bridge covariates model
HDOP: Horizontal dilution of precision
MCMC: Markov chain monte carlo
ODBA: Overall dynamic body acceleration
UD: Utilization distribution
